# Revolutionizing Cardiac Imaging: A Scoping Review of Artificial Intelligence in Echocardiography, CTA, and Cardiac MRI

**DOI:** 10.3390/jimaging10080193

**Published:** 2024-08-08

**Authors:** Ali Moradi, Olawale O. Olanisa, Tochukwu Nzeako, Mehregan Shahrokhi, Eman Esfahani, Nastaran Fakher, Mohamad Amin Khazeei Tabari

**Affiliations:** 1 Internal Medicine, HCA Florida, Blake Hospital, Morsani College of Medicine, University of South Florida, Bradenton, FL 34209, USA; 2 Center for Translational Medicine, Semmelweis University, 1428 Budapest, Hungary; 3 Internal Medicine, Adjunct Clinical Faculty, Michigan State University College of Human Medicine, Trinity Health Grand Rapids, Grand Rapids, MI 49503, USA; 4 Internal Medicine, Christiana Care Hospital, Newark, DE 19718, USA; 5 School of Medicine, Shiraz University of Medical Sciences, Shiraz 71348-45794, Iran; 6 Faculty of Medicine, Semmelweis University, 1085 Budapest, Hungary; 7 Student Research Committee, Mazandaran University of Medical Sciences, Sari 48175-866, Iran

**Keywords:** artificial intelligence, echocardiography, cardiac imaging, magnetic resonance imaging

## Abstract

Background and Introduction: Cardiac imaging is crucial for diagnosing heart disorders. Methods like X-rays, ultrasounds, CT scans, and MRIs provide detailed anatomical and functional heart images. AI can enhance these imaging techniques with its advanced learning capabilities. Method: In this scoping review, following PRISMA (Preferred Reporting Items for Systematic Reviews and Meta-analyses) Guidelines, we searched PubMed, Scopus, Web of Science, and Google Scholar using related keywords on 16 April 2024. From 3679 articles, we first screened titles and abstracts based on the initial inclusion criteria and then screened the full texts. The authors made the final selections collaboratively. Result: The PRISMA chart shows that 3516 articles were initially selected for evaluation after removing duplicates. Upon reviewing titles, abstracts, and quality, 24 articles were deemed eligible for the review. The findings indicate that AI enhances image quality, speeds up imaging processes, and reduces radiation exposure with sensitivity and specificity comparable to or exceeding those of qualified radiologists or cardiologists. Further research is needed to assess AI’s applicability in various types of cardiac imaging, especially in rural hospitals where access to medical doctors is limited. Conclusions: AI improves image quality, reduces human errors and radiation exposure, and can predict cardiac events with acceptable sensitivity and specificity.

## 1. Introduction

Imaging is the most common diagnostic tool in the field of cardiology, showing promising outcomes in diagnosis. While X-rays were once the sole imaging tool available for diagnosing cardiac abnormalities, there are now more options, including echocardiography, angiographic imaging, various types of computed tomography (CT) scans, and cardiac magnetic resonance (CMR) imaging. These imaging methods provide valuable information about the heart, including accurate morphology, function, coronary vessel perfusion, and more [1,2]. Diagnostic cardiac imaging has seen significant growth and usage over the years. For example, radiologists’ non-invasive coronary computed tomography angiography (CTA) rate has increased remarkably, reflecting a growing reliance on advancing imaging techniques for cardiovascular disease assessment and risk stratification [3] The capability of Artificial intelligence (AI) is determined by its machine learning (ML) algorithms, which possess the capacity to acquire knowledge from past data and enhance their performance through experience and training. Deep learning (DL) is a branch of ML that can generate data using artificial neural networks without relying on pre-existing data [4]. AI’s role in cardiology and cardiac imaging is growing, and it has the potential to transform the way patient care is provided fundamentally. A recent study has shown a 16% discordance between local doctors and specialized radiologists/cardiologists, with local doctors overestimating severe artery narrowing (>50%). This study shows a gap in diagnoses and proves to be one of the many aspects where AI integration could assist in mitigating human errors and in the accuracy of diagnostic cardiac imaging analyses [5]. Cardiovascular disease-related deaths have risen to be among the leading causes of death in the past decade. Therefore, it is crucial to advance medical processes further to increase efficiency and accuracy and predict cardiac events. Studies have shown that AI algorithms perform well in diagnosing, predicting, and stratifying cardiovascular diseases (CVDs) and could even assist in the early diagnosis of certain cardiac diseases during routine primary care [6,7]. AI algorithms can aid healthcare workers in identifying, diagnosing, and forecasting the prognosis of CVDs. Integrating AI technology into cardiac imaging instruments offers substantial advantages to the medical industry, including enhanced precision, reduced expenses, and shorter procedure durations [8]. This scoping review aims to review data provided by multiple studies that have integrated various AI algorithms and software to predict and classify different CVDs in comparison to clinician assessments using medical imaging methods such as CT, CMR, and echocardiography. The review will assess the accuracy, specificity, and sensitivity of AI, exploring its potential to revolutionize cardiac imaging and its prospects for integration into the diagnostic cardiac imaging process.

## 2. Method

A comprehensive literature review was conducted till 16 April 2024 to identify pertinent articles from 3679 articles, from which 3584 remained for identification after the duplicated articles were excluded from the databases PubMed, Scopus, web of Science, and Google Scholar. In the search strategy, two primary subgroups of keywords and Medical Subject Headings (MeSH) were utilized. The first subgroup comprised terms associated with AI and mechanic intelligence, and the second subgroup included terms related to cardiac imaging, cardiac MRI, cardiac ultrasound, cardiac sonography, and cardiac angiography (Table 1). The search methodology has been adapted to the query format specific to each database. We have included studies that meet the criteria for inclusion in our research. The review team has independently undertaken all steps, and any disagreement has been resolved in consultation with them. Our methodology follows the PRISMA guidelines (Preferred Reporting Items for Systematic Reviews and Meta-analyses) [9].

### 2.1. Inclusion and Exclusion Criteria

For studies to be included in this scoping review, the articles must fit into the following criteria:

(1) To mitigate the possible confounding effect of any intervention, observation methods are used.
(2) Included for consideration were original studies, all types of cardiac imaging modules, and studies from all countries, which were in English.
(3) Excluded from consideration were studies that were review papers, animal studies, populations without a heart condition, case reports, case series, other languages that were not English, articles that were only abstract and lacked full text, and grey literature.

### 2.2. Data Extraction and Study Quality Assessment

The eligibility for inclusion in this scoping review is determined by the title and abstract of each study, which two independent evaluators determined. Excluded were studies that did not meet our criteria. All other studies have been thoroughly examined, and the data extraction process was carried out solely on those that meet these criteria.

## 3. Result

**Figure 1:**
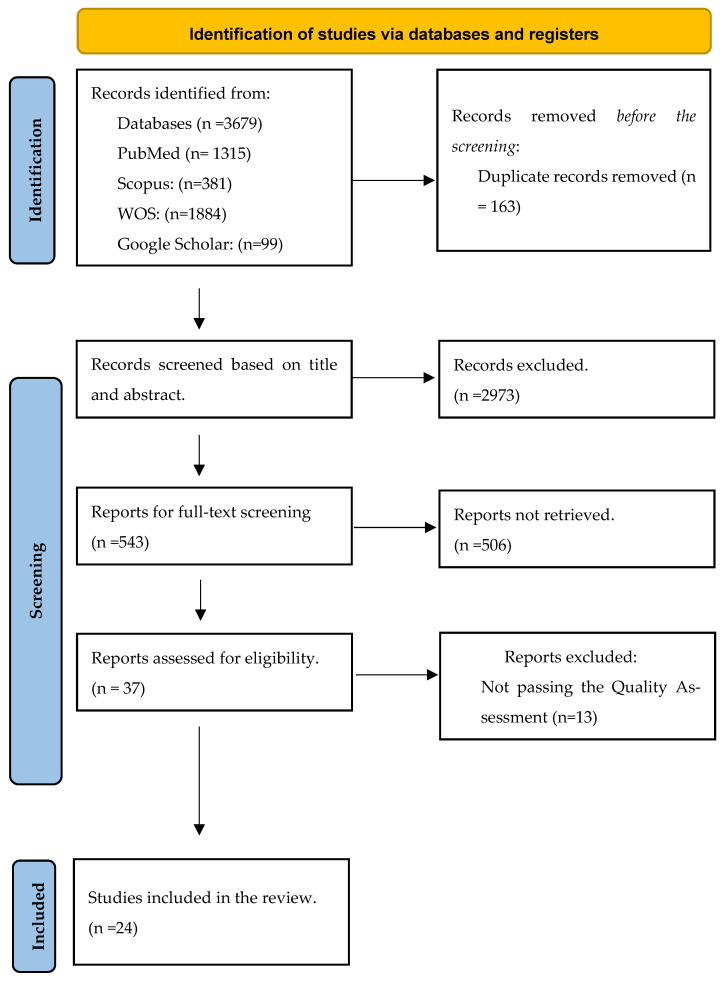
PRISMA guidelines (Preferred Reporting Items for Systematic Reviews and Meta-analyses).

Different databases, including PubMed, Scopus, Web of Science, and Google Scholar, were used with a search strategy outlined in Table 1. A total of 3679 articles were extracted for screening. After removing 163 duplicates using EndNote and manual methods, 3516 papers were selected for title and abstract screening. The screening focused on patients with cardiac abnormalities who needed echocardiography, CT scans for the heart, or CMR imaging, where AI was used for cardiac imaging methods. Two groups of independent authors reviewed the titles and abstracts, and 543 articles were selected for full-text evaluation based on the type of cardiac disorder, the implementation of AI, and the comparison between using AI and not using AI. After quality assessment with the Newcastle-Ottawa Quality Assessment Checklist (for case-control and cohort studies), 24 articles were selected to be included in our study. This scoping review followed the PRISMA 2020 guidelines, as you can see in Figure 1 [9].

**Figure 2:**
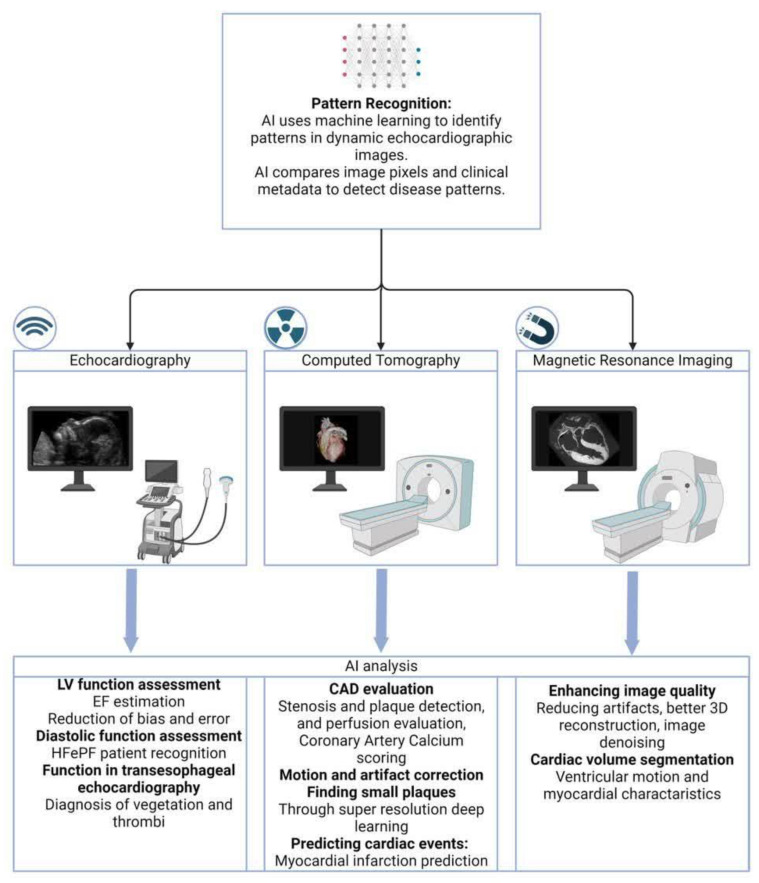
Artificial intelligence (AI) applications in cardiac imaging.

Figure 2 shows a brief review of how AI can be applied in different types of cardiac imaging, including echocardiography, CT angiography, and Cardiac MRI.

The use of AI with DL capabilities is significant in cardiology. In a study involving 3407 participants, AI’s application in echocardiography demonstrated its potential across a wide spectrum of diseases, including heart failure, ischemic heart disease, coronary artery disease (CAD), systolic function abnormalities, valvular abnormalities, and acute coronary syndrome. Deep learning models (DLMs) can aid in predicting future cardiac events, accelerating imaging processes, and improving diagnostic accuracy. Table 2 provides a brief review of studies exploring AI’s role in echocardiography.

The use of AI in CTA can revolutionize the field by reducing exposure time, predicting the risk of ischemia in patients with CAD, and demonstrating high sensitivity and specificity in detecting lesions in coronary arteries. Table 3 provides a brief review of the impact of AI in CTA.

Table 4 provides an overview of the impact of AI in cardiac MRI across various diseases, including RV abnormalities, cardiomyopathies, and cardiac structure and function abnormalities. AI can significantly transform CMR methods by reducing the time needed to process images, improving quality, and offering precision that can surpass human capabilities.

Newcastle-Ottawa Quality Assessment Checklist (for case-control and cohort studies) provides a comprehensive evaluation of various studies conducted by different authors. The checklist assesses the quality of each study based on three primary criteria, including Selection, Comparability, and Outcome, and provides an overall rating.

The “Selection” criterion evaluates the adequacy of case definition, representativeness of the cases, selection of controls, and definition of controls. Most of the studies in the table received a score of 3 out of a possible 4. The study by Kang et al. stands out with a perfect score of 4 in this category [14]. For the “Comparability” criterion, which assesses the comparability of cases and controls based on the design or analysis, the majority of studies scored 1 out of a possible 2. Only a few studies, specifically those by Pandey et al. [11], Park et al. [16], and Paul et al. [19], received the highest score of 2, indicating better comparability. In the “Outcome” criterion, which looks at the assessment of outcome and follow-up period for cohort studies, all studies consistently scored the maximum of 3, indicating a high level of outcome assessment quality across the board.

Overall, every study listed in the table is rated as “good,” signifying a general consensus of high quality according to the Newcastle-Ottawa Scale (Table 5).

## 4. Discussion

### 4.1. AI Patterns in Cardiac Imaging

**Figure 3:**
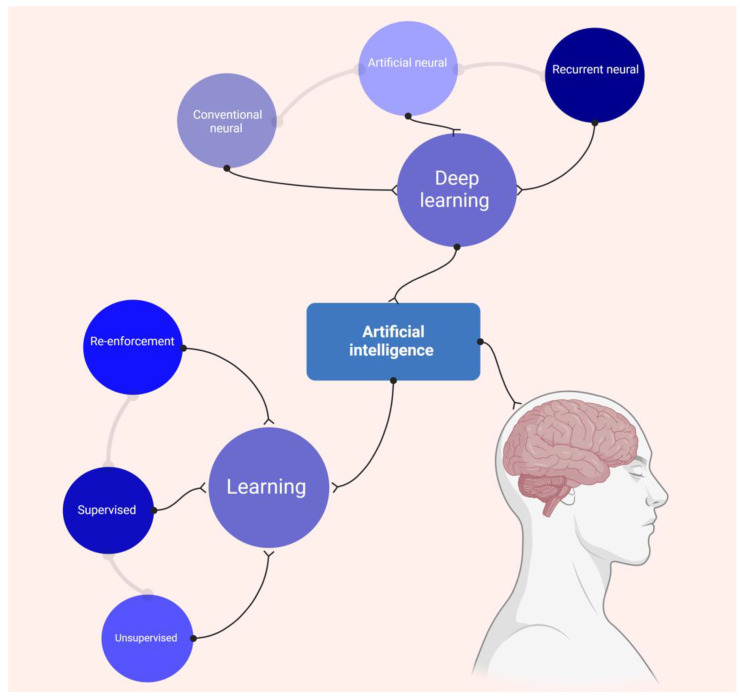
AI classification and subgroups.

AI is a revolving tool that has the capability to learn and do tasks that require human intelligence. ML forms the core of learning abilities and is categorized into three types: supervised, unsupervised, and re-enforced. To forecast future events in supervised and unsupervised AI, they use previously categorized and uncategorized data, respectively. Reinforcement AI works in conjunction with environmental sensors, such as cameras or GPS, to perform robotic interventions. DL is another ML class requiring higher features, such as neural networks, like the human brain. This artificial neural network can read, learn, and transform input data into compounded output data. It is classified as artificial neural, convolutional neural, and recurrent neural. Artificial neural networks perform the task of analyzing and processing information in a manner like the human brain (Figure 3). Convolutional neural networks can analyze visual pictures, while recurrent neural networks may establish connections between nodes in a directed graph [34,35].

A detailed and relevant database is valuable for AI; however, ML can enrich the database by breaking the data into pieces of information. For example, brightness, pixel density, clinical reports, etc., can be used in imaging. ML uses its learning and DL abilities to create a representative model [36,37]. Anatomical structures such as valves, ventricles, or coronary vesicles primarily relate to cardiac abnormalities, and measuring various parameters can aid in their diagnosis. With DL abilities, AI can process content extraction from input medical images, called image segmentation, to produce 2D and 3D meshes [37,38]. Image segmentation relies on various landmarks, such as well-defined anatomical points. With deep reinforcement learning, AI is able to not only detect these landmarks but also learn how to localize them. For example, this method has been tested on 5000 CT scans with complete accuracy in detecting cardiovascular landmarks in less than a second [39]. Image segmentation and DL have also been recently used in CMR images [40]. The type of ML depends on the kind of feedback the algorithms receive during the learning process. When used for a specific goal, it is crucial to understand each category’s unique future [8].

### 4.2. AI Applications in Echocardiography

Echocardiography is a handy imaging diagnostic tool that allows for real-time imaging and can detect various abnormalities. Echocardiography can provide a 2D heart image, but it might be challenging to read, and doctors might have different diagnoses. AI and other ML tools can help identify multiple patterns in the echocardiography [41,42]. AI’s ability is based on learning, and it might be more challenging to train AI for the pattern of echocardiography as they are moving subjects rather than a single picture like CT or MRI images. AI can compare pixels in an image and use clinical metadata to find a specific disease pattern based on that information. Combining AI algorithms and clinical databases can improve the accuracy of echocardiography and reduce the chance of variability in diagnosis among medical doctors [42]. One critical role of echocardiography is assessing left ventricle (LV) function. The ejection fraction (EF) is one of the essential values for estimating LV function. However, estimating EF traditionally involves manual measurement of end-diastolic and end-systolic volumes, which can be subject to significant variability and bias. Automating echocardiographic measurements with AI offers the potential for more accurate and efficient assessments. By employing ML algorithms, AI can analyze echocardiographic images and calculate EF with greater precision and speed than manual methods. This automated approach reduces the risk of measurement errors and bias, leading to more reliable clinical evaluations of LV function [8]. In a study, researchers compared automated echocardiographic measurement with the manual method of calculating EF from 2D images. The correlation was similar when the image quality was excellent or moderate; however, the correlation was slightly worse when the quality was poor [43]. Echocardiography in heart failure patients with preserved ejection fraction (HFpEF) can be challenging yet crucial for evaluating diastolic function. Based on their echocardiography results, up to one-third of HFpEF patients reportedly exhibit normal diastolic function. Leveraging AI capabilities, such as those used in measuring systolic function, could offer a novel approach to estimating diastolic function [8,44]. In a study conducted by Pandey et al. using ML, they developed a model to assess diastolic function. They compared it with the American Society of Echocardiography (ASE) 2016 diastolic guidelines grading system. Their model demonstrated a higher receiver-operating characteristic (ROC) value than the ACE 2016 guidelines [11]. Echocardiography has the potential to give a considerable amount of diagnostic information about the heart. Still, it takes time for experts to evaluate it quickly during a routine echocardiography. AI can analyze that information much faster than human capability. This information can give us insight into various diseases, such as coronary disease, cardiomyopathies, valvular heart disease, and many more [8]. Various types of echocardiography, such as transesophageal echocardiography, can diagnose intracardiac masses such as thrombosis, tumors, and vegetation. Using AI-aided transesophageal echocardiography, researchers looked at how AI can help find left atrial thrombi in patients with atrial fibrillation. They discovered that the AI-derived algorithm made the results much more accurate than just using transesophageal echocardiography [45].

### 4.3. AI and CT Imaging in Cardiology

CAD is the leading cause of mortality, making it essential for physicians to evaluate the coronary vessels [46]. CTA is significant in evaluating stenosis, assessing plaque, analyzing myocardial perfusion, and determining the coronary artery calcium score (CAC). By incorporating AI into coronary CT, the accuracy and efficiency of these procedures can be enhanced, leading to improved quality CT scan pictures through reconstruction, motion correction, and reduced radiation exposure [47,48]. CAC scoring is an inexpensive and highly efficient technique. The CAC score independently predicts major cardiac events (MACs) [49]. AI can significantly impact the CAC, reducing the need for radiologists to analyze the data. In 2007, researchers conducted a study to evaluate AI’s sensitivity in detecting and predicting coronary calcification on 76 non-contrast enhanced ECG-gated CT scans. The AI algorithms could detect 73.8% of cases [50]. Semi-automated and fully automated segmentation methods significantly improve the deployment of AI for CAC analysis. An automatic segmentation AI was employed to assess CAC in a study involving 1793 patients who received non-contrast CT scans for lung cancer. These scans were performed without ECG-gating, which is necessary for estimating CAC. The κ coefficient exhibited excellent reliability (0.85) in comparing the automated and reference scores for Agatston risk groups [51]. In general, using CACs combined with AI can decrease human errors and enhance the reliability of result prediction [48]. The other way that AI can help is motion correction; motion artifacts in coronary artery CT scans result in distorted images and typically occur when the patient has an irregular or elevated heart rate. To address this problem, several non-AI (augmented with ECG) and AI software solutions have been created, achieving accuracy rates of 81.5–96.8% and 81–85%, respectively. This study has shown that additional evaluations are required for the clinical implementation of the AI software [48,52]. CTA is a non-invasive diagnostic tool for diagnosing CAD. However, this method differs from other diagnostic tools due to the thinner slice thickness, which can lead to low-quality images. Deep learning reconstruction (DLR) algorithms can help decrease the image quality by reducing the noise. Multiple studies have shown that this DLR algorithm significantly reduces the image noise, improves the quality, and reduces radiation exposure [48,53,54]. A new super-resolution deep learning reconstruction (SR-DLR) technique has emerged recently. This technique has a lower exposure rate and a higher noise level, but it can learn to improve resolution and reduce image noise. Compared to DLR, SL-DLR can provide better visualization of small atherosclerosis plaques [48,55]. Many studies have shown that using AI in cardiac CT scans can help predict cardiac events. In a study conducted by Lin et al., it was found that by using DL, they were able to accurately predict the risk of myocardial infarction [56]. Nakanishi et al. demonstrated that a ML model, which integrates many factors, effectively forecasted fatalities associated with cardiovascular disease [57].

### 4.4. AI and Cardiac MRI

CMR generally requires a long time, 45–60 min, and requires breath-holding in some parts of the procedure to capture and then segment and analyze the image. AI can improve CMRs’ accuracy, reproducibility, and precision [58]. Researchers have begun employing AI to automate the CMR imaging process to enhance the efficiency of MRI analysis. These AI processes in CMR are classified into various categories, such as reconstruction, enhancement, and denoising. Multiple research studies have employed AI capabilities to generate three-dimensional (3D) representations of the heart. These studies have utilized AI’s neural network to expedite the process of reconstructing 3D images of the entire heart [47]. MRI signals are the primary data for conducting CMR imaging to collect heart images. However, these signals are subjected to undersampling during this process, decreasing the signal-to-noise ratio. Using AI-accelerated approaches in cardiac MRI can help reduce the problem of undersampling. DL can acquire these processes using Convolutional Neural Networks, the main techniques used in CMR by DL systems. Integrating AI and compressed CMR reduces undersampling artifacts and improves the efficiency and accuracy of reconstructing cardiac images. These innovations have great potential for enhancing diagnostic capabilities and patient care [59,60]. Another function of AI in cardiac MRI can be conceptualized as enhancement. AI can enhance the clarity and sharpness of photographs of poor quality. Enhancing the resolution of low-quality photos can significantly decrease scanning time by reducing the duration of the scanning process. This system uses adversarial network-based models and utilizes its learning capabilities to assess the probability of generating a more realistic image, surpassing a mere resolution enhancement generator [59]. In cardiac MRI, imaging denoising can also help improve the SNR. Traditional methods can blur the images. There are many challenges in using AI to denoise the images. Due to multi-coil arrays and nonlinear image reconstruction that impact image noises, denoising by AI is much more complicated and needs more study in this field [59,61]. Cine-MRI offers insights into the dynamics of ventricular wall motion, the myocardium’s thickness, and the ventricles’ volume [47]. Cardiac volume segmentation is crucial for assessing biventricular function and involves tracing the contours of the endocardium and epicardium [62]. This technique is time-consuming. Various AI models have been created to aid in this segmentation process, enhancing its speed and precision [47,63]. CMR can non-invasively provide detailed information about the characteristics of heart tissue. The T1 and T2 mapping techniques, along with late gadolinium enhancement (LGE), can be used to detect an enlarged extracellular compartment in diseases such as amyloidosis and measure myocardial edema. These techniques provide valuable information regarding both focal and diffuse cardiac diseases [47]. According to Chang et al., the DL algorithm effectively segmented the myocardium in 99.3% of slices in the native T1 map and 89.8% of slices in the post-T1 map [64]. Using AI in CMR has the capability to automate the analysis of pictures and help in measurements. Advanced AI has the ability to reduce the duration of scanning, image reconstruction, and data analysis processes. While there may still be some lingering concerns about analysis oversight, there is no reason to be afraid of using ML in CMR. ML relies on the process of learning and training. Therefore, these systems should help thoroughly analyze patients to improve comprehension. This will enable models to provide insights into causality and offer personalized counsel that can be acted upon for each unique patient [59].

### 4.5. Limitation of Using AI in Cardiac Imaging

While AI has numerous advantages in healthcare, especially in cardiac imaging, it also has certain limits. It is essential to comprehend that AI should be employed to reduce or eradicate errors rather than expand them [65]. Another issue regarding the utilization of AI is the potential professional liability of physicians in the event of an erroneous decision. For instance, a novice physician may unquestioningly rely on the AI’s erroneous determination based on algorithms [66,67] AI algorithms are primarily created in university-affiliated hospitals, which may not accurately represent the entire population. Consequently, it is challenging to generalize these algorithms. Nevertheless, this problem can be mitigated by providing a more extensive database from the general population for AI models and algorithms [68,69]. These are some limitations regarding the use of AI in cardiac imaging. Although this review provides a good overview of the subject across various types of cardiac imaging, more studies are needed to address specific questions, such as the exact sensitivity and specificity of each imaging method when applied with AI. It is important to understand that the goal of using AI is to assist physicians by reducing errors and improving patient care rather than increasing errors, which can have significant downside effects on healthcare.

### 4.6. Current Challenges and Gaps

The performance of AI algorithms heavily relies on the quality and quantity of the training data. Variability in imaging protocols, equipment, and patient populations across different institutions can lead to inconsistent results. Standardizing imaging protocols and creating large, diverse, and annotated datasets are essential to improve the generalizability and robustness of AI models [70]. AI models, particularly deep learning algorithms, often function as “black boxes,” providing little insight into how decisions are made. This lack of interpretability can hinder clinical adoption, as healthcare providers need to understand and trust AI-driven recommendations. Developing models with improved transparency and explainability is crucial for gaining clinician trust and facilitating the integration of AI into clinical workflows [71]. While AI has demonstrated impressive capabilities in cardiac imaging, integrating these technologies into existing clinical workflows remains a challenge. Seamless integration requires not only technical compatibility but also consideration of clinical workflow dynamics, user training, and acceptance. Future research should focus on developing user-friendly interfaces and workflows that enhance rather than disrupt existing practices. The implementation of AI in medical imaging raises several ethical and legal issues, including data privacy, informed consent, and accountability. Establishing clear regulatory frameworks and guidelines is essential to address these concerns and ensure the ethical use of AI in healthcare [72]. Large-scale, multi-center clinical trials are necessary to validate the efficacy and safety of AI algorithms in diverse clinical settings. These studies should assess not only diagnostic accuracy but also the impact on clinical outcomes, workflow efficiency, and cost-effectiveness [73]. The Newcastle-Ottawa Scale is a valuable tool for assessing the quality of non-randomized studies, providing a systematic approach to evaluate selection, comparability, and outcome. However, the studies listed in this research, while uniformly rated as “good,” exhibit limitations such as potential selection bias, limited comparability control, and lack of nuanced grading. Researchers and reviewers should be aware of these limitations and consider supplementary quality assessment methods to ensure a comprehensive evaluation of study quality.

## 5. Conclusions

The integration of AI into cardiac imaging offers significant advancements in image quality, diagnostic accuracy, and procedural efficiency. Our review demonstrates that AI algorithms, particularly deep learning models, can match or exceed the performance of experienced cardiologists in various cardiac conditions. However, the transition from controlled studies to real-world clinical practice presents challenges, including data variability, ethical considerations, and the need for extensive validation. Future research should focus on robust validation studies, practical implementation strategies, and ensuring equitable access to AI technologies. Addressing these challenges will enable AI to enhance cardiac care and improve patient outcomes globally.

## Figures and Tables

**Table 1:** Search Strategy and Databases.

Databases	Mesh Term	Result	Date
PubMed	((((Artificial intelligence[MeSH Terms]) OR (machine intelligence[MeSH Terms])) OR (AI[Title/Abstract])) OR (cognitive computing[Title/Abstract])) OR (robotic intelligence[Title/Abstract]) AND ((((cardiac imaging[MeSH Terms]) OR (cardiac ultrasound[MeSH Terms])) OR (cardiac MRI[MeSH Terms])) OR (angiography[MeSH Terms])) OR (cardiac US[MeSH Terms])	1315	16 April 2024
SCOPUS	(artificial AND intelligence OR machine AND intelligence OR ai OR cognitive AND computing OR robotic AND intelligence) AND (cardiac AND imaging OR cardiac AND MRI OR cardiac AND ultrasound OR angiography OR cardiac AND sonography OR heart AND sonography)	381	16 April 2024
Web of Science	(ALL = (Artificial intelligence OR machine intelligence OR AI)) AND ALL = (cardiac imaging OR cardiac MRI OR Cardiac US OR cardiac ultrasound) and Article (Document Types)	1884	16 April 2024
Google Scholar	(Artificial intelligence OR machine intelligence OR AI OR cognitive computing OR robotic intelligence) AND (cardiac imaging OR cardiac MRI OR Cardiac US OR cardiac ultrasound OR cardiac sonography)	99	16 April 2024

**Table 2:** Use of AI in echocardiography.

Author, Year.	Number of Participants	Cardiac Abnormality	Type of DLM	Result	Conclusion
Kusunose et al., 2021 [10].	400	Regional wall motion abnormalities	Deep convolutional neural network	DLM algorithm showed comparable performance to cardiologists in detecting WMAs: AUC of 0.99 vs. 0.98 (*p* = 0.15).	Deep convolutional neural network can be Use to diagnose myocardial ischemia with echocardiography.
Pandey et al., 2021 [11].	1242	Heart failure with preserved EF	1. unsupervised clustering approach (topological data analysis network) 2. cloud-based automated ML	The AUC for predicting elevated left ventricular filling pressure is higher compared to the 2016 ASE guidance grades (0.88 vs. 0.67; *p* = 0.01).	The neural network. classifier in this study provides a practical solution to overcome the limitations of current clinical standards in accurately characterizing the burden of LVDD in HFpEF.
Salte et al., 2021 [12].	200	Various cardiac pathologies (measuring LV function)	Different type of artificial neural networks (ANN) 1. recurrent ANN architecture 2. U-net architecture	-The AI method accurately classified all apical views and timed cardiac events in 89% of patients. -Mean GLS was −12.1% ± 5.0% (AI) and −13.5% ± 5.3% (conventional).	DL networks eliminate manual tracing, increasing efficiency and reproducibility. Fully automated AI measurements facilitate the implementation of GLS in clinical practice.
Upton et al., 2022 [13].	578	Severe Coronary artery disease	convolutional neural network.	-Using AI to identify severe coronary artery disease achieved 92.7% specificity and 84.4% sensitivity with cross-fold validation. -The AI tool increased inter-reader agreement, confidence, and disease detection sensitivity by 10%, with an AUC of 0.93.	Automated analysis of stress echocardiograms is possible using AI. Providing automated classifications to clinicians could improve accuracy, inter-reader agreement, and reader confidence when reading stress echocardiograms.
Kang et al., 2023 [14].	9	Systolic function abnormality	U-Net and new AI model based on U-Net	U-Net had an AUC of 0.982, 0.996, 0.983, 0.996, and 0.992 in each fold, while the new model achieved AUCs of 0.994, 0.998, 0.997, 0.997, and 0.994.	Compared to U-Net, the proposed model demonstrated better performance across all metrics, enabling more accurate segmentation of the LV for detailed evaluation of the heart’s systolic function during CPR.
Krishna et al., 2023 [15].	256	Aortic stenosis	ANN (Us2.ai)	AI demonstrated strong correlation with human measurements in: -Aortic valve peak velocity (r = 0.97, *p* < 0.001) -Mean pressure gradient (r = 0.94, *p* < 0.001) -Stroke volume index (r = 0.79, *p* < 0.001)	This AI technology could reduce interscan variability, enhance AS interpretation and diagnosis, and enable accurate and reproducible management of AS patients.
Park et al., 2023. [16].	395	Acute coronary syndromes	-MD-CTA model. -standard convolution neural network	The novel DL model showed significantly improved AUC, sensitivity, and specificity compared to the convolution neural network for patient-level prediction: AUC (0.899 vs. 0.724), sensitivity (87.1% vs. 71.0%), and specificity (85.3% vs. 68.0%).	This new DL model shows promise in identifying atherosclerotic plaque erosion using non-invasive coronary CTA images and significantly outperforms experienced cardiologists
Salte et al., 2023 [17].	Dataset I: n = 40 Dataset II: n = 32	LV GLS	ANN	-The AI method correctly classified the view in 96% (231/240) of recordings in dataset I and 97% (187/192) in dataset II. -AI accurately classified cardiac events (end diastole, systole, and end systole) in 99% (238/240) of recordings in dataset I and 97% (187/192) in dataset II.	This AI method, with automated LV GLS measurements, reduced test-retest variability, eliminated reader bias, and can facilitate LV GLS implementation, improving workflow in clinical echocardiography.
Zamzmi et al., 2023 [18].	255	Estimation of RAP by IVC measurement during echocardiography	Conventional ML models	Strong agreement (r = 0.96) between automatically calculated and manually measured IVC values.	-Fully automated and cost-effective tool for analyzing dIVC and cIVC. -Enhances clinical decision-making through quantitative data insights. -Potential to improve patient outcomes through informed medical interventions.

AI: artificial intelligence, WMAs: wall motion abnormalities, AUC: area under the receiver-operating characteristic curve, LVDD: left ventricular diastolic Dysfunction, HFpEF: heart failure with preserved ejection fraction, GLS: global longitudinal strain, LV: left ventricle, ASE: American Society of Echocardiography, LOA: limits of agreement, ANN: artificial neural networks, AS: Aortic stenosis, CTA: computed tomography angiography, DL: deep learning, RAP: right atrial pressure, IVC: inferior vena cava, ML: machine learning, dIVC: Diameter of inferior vena cava, cIVC: Collapsibility index of inferior vena cava, CNN: convolutional neural network.

**Table 3:** Use of AI in cardiac CTA.

Author, Year	Number of Patients	Cardiac Abnormality	Type of Deep Learning	Result	Conclusion
Paul et al., 2022 [19].	53	Coronary artery disease	neural network	Accuracy of the DLM was 96%.	Accuracy was excellent for differentiating patients with vs. without stenoses ≥50%.
Andre et al., 2023 [20].	120	Coronary artery disease	Siemens Automatic Coronary Analysis	The time for the coronary CTA assessment was reduced in the human AI group vs. standard group by approximately 27%.	AI-based analysis significantly improves clinical by reducing CTA analysis reporting time without compromising diagnostic accuracy.
Cobo et al., 2023 [21].	658	Coronary artery tortuosity (CAT)	convolutional neural network	DLM Vs expert radiological visual examination for detecting CAT, with a sensitivity of 87 ± 10% versus 84 ± 2% and a specificity of 88 ± 10% versus 86 ± 4%	DLM could have a beneficial impact on preventing cardiac complication, shortening coronary angiography examination times.
Dey et al., 2018 [22].	254	Coronary artery disease	supervised learning model usable in predicting revascularization after ischemia.	Results suggest that ML approach may outperform conventional statistical integration of the same data	The Integrated ML ischemia risk score improved the prediction of lesion-specific ischemia.
Dundas et al., 2023 [23].	120	Coronary artery disease	AI-based coronary stenosis quantification (AI-CSQ) software V1.	The AI-CSQ tool demonstrated a sensitivity of 95%, specificity of 57%, and accuracy of 76%. On a per-vessel basis, sensitivity was 90%, specificity 76%, and accuracy 80%.	AI-based coronary stenosis quantification at coronary CT angiography shows high diagnostic performance and sensitivity for stenosis detection compared to quantitative coronary angiography, both per-patient and per-vessel.
Griffin et al., 2023 [24].	303	Coronary artery disease	convolutional neural network models.	Per-patient for detecting ≥50% stenosis sensitivity (94%,), specificity (68%), positive predictive value (81%), negative predictive value (90%), and accuracy (84%) and for detecting ≥70% stenosis were 94%, 82%, 69%, 97%, and 86%, respectively.	AI-based evaluation demonstrated high diagnostic performance for the identification, exclusion, discrimination, and correlation to a quantitative coronary angiography reference standard.
Han et al., 2022 [25].	196	Coronary Artery Disease	Automated AI algorithms.	The AI system demonstrated 94% sensitivity at the patient level and 78% sensitivity at the vessel level. These sensitivity rates were higher than those of non-AI assisted readings by two cardiovascular radiologists.	using AI increased the sensitivity of inexperienced readers and improved the consistency of coronary stenosis diagnosis via coronary CT angiography.
Ihdayhid et al., 2022 [26].	1849	Coronary artery calcium score	three-dimensional (3D) fully convolutional neural network.	The AI-based fully automated coronary artery calcium scoring model accurately detected coronary artery calcium, closely aligning with human readings, and demonstrated comparably lower analysis times.	fully automated coronary artery calcium (CAC) scoring model shows high accuracy and low analysis times.
Zhang et al., 2024 [27].	1801	Coronary artery disease.	Convolutional Neural Networks	Observed greatly improved efficiency, and maintains high diagnostic accuracy and the effectiveness in stratifying patients for cardiovascular events.	Fully automated AI-based coronary CT angiography significantly improves workflow efficiency compared to the semi-automated mode.
Assen et al., 2020 [28].	Cohort one: 95 Cohort two: 168	Coronary artery calcium score	Deep-learning convolution neural and a fully connected neural network.	Deep-learning-based automated calcium quantification on chest CT shows excellent correlation with manual calcium volume quantification and Agatston scores from cardiac CTs.	Automated analysis can increase workflow efficiency and help manage the growing number of acquisition requests, assisting radiologists.
Yoneyama et al., 2019 [29].	59	Coronary artery disease	artificial neural network.	1. Observer A detected CAD with 83.6% accuracy in the RCA, 89.3% in the LAD, and 94.4% in the LCX. 2. Observer B achieved 72.9% accuracy in the RCA, 84.2% in the LAD, and 89.3% in the LCX. 3. The artificial neural network (ANN) had 79.1% accuracy in the RCA, 89.8% in the LAD, and 89.3% in the LCX.	AI diagnoses are comparable to those by nuclear medicine physicians.

DLM: deep learning model, AI: artificial intelligence, CTA: Computed Tomography Angiography, CAT: Coronary artery tortuosity, ML: machine learning, AI-CSQ: AI-based coronary stenosis quantification, CT: Computed Tomography, coronary artery calcium (CAC) scoring, CAD: coronary artery disease, RCA: right coronary artery, LAD: left coronary artery, LCX: Left Circumflex Artery, ANN: artificial neural network.

**Table 4:** Use of AI in Cardiac MRI.

Author, Year	Number of Patients	Cardiac Abnormality	Type of Deep Learning	Result	Conclusion
Åkesson et al. 2023 [30].	1434	Right ventricular. abnormalities	Convolutional Neural Networks	The average time reduction using DLM was 5 min and 17 s.	A DL-based model can sufficiently make the right ventricular assessments faster.
Cau et al. 2024 [31].	107	Ischemic and non-ischemic cardiomyopathy	gradient boosting generalized additive model (GB-GAM)	GB-GAM had The Sensitivity of 0.72 and Specificity of 0.68 (AUC = 0.82) in discriminating between ICM and NICM.	DLM can discriminate between CM and NICM with high accuracy reducing cost and time of the examination.
Davies et al. 2022 [32].	109	Cardiac structure and function abnormalities	Convolutional neuronal network	Measuring left ventricular metrics had precision of 0.94–0.95	DLM was faster and more precision. Compare to human performance.
Zhang et al. 2019 [33].	212	Coronary artery disease and MI	1. convolutional neural network 2. recurrent neural network	Using DLM in nonenhanced cardiac MRI has per-segment Sensitivity of 89.8% And Specificity of 99.1% in detecting chronic MI.	Using DLM in nonenhanced cardiac cine MRI can find the likely location of chronic MI

DLM: deep learning model, DL: deep learning, AUC: area under the receiver-operating characteristic curve, ICM: ischemic cardiomyopathies, NICM: non-ischemic cardiomyopathies, MI: myocardial infarction, MRI: Magnetic Resonance Imaging.

**Table 5:** Newcastle-Ottawa Quality Assessment Checklist (for case-control and cohort studies).

Author	Selection	Comparability	Outcome	Overall
Kusunose et al. [10]	3	1	3	good
Pandey et al. [11]	3	2	3	good
Salte et al. [12]	3	1	3	good
Upton et al. [13]	3	1	3	good
Kang et al. [14]	4	1	3	good
Krishna et al. [15]	3	1	3	good
Park et al. [16]	3	2	3	good
Salte et al. [17]	3	1	3	good
Zamzmi et al. [18]	3	1	3	good
Paul et al. [19]	3	2	3	good
Andre et al. [20]	3	1	3	good
Cobo et al. [21]	3	1	3	good
Dey et al. [22]	3	1	3	good
Dundas et al. [23]	3	1	3	good
Griffin et al. [24]	3	1	3	good
Han et al. [25]	3	1	3	good
Ihdayhid et al. [26]	3	1	3	good
Zhang et al. [27]	3	1	3	good
Assen et al. [28].	3	1	3	good
Yoneyama et al. [29]	3	1	3	good
Åkesson et al. [30]	3	1	3	good
Cau et al. [31]	3	1	3	good
Davies et al. [32]	3	1	3	good
Zhang et al. [33]	3	1	3	good

## Data Availability

The datasets generated during the current study are not publicly available but are available from the corresponding author upon reasonable request.
